# Conceptual proposal for LLM-generated FDG PET/CT follow-up reports in melanoma: a pilot study on model stability and blinded expert evaluation

**DOI:** 10.3389/fnume.2026.1723650

**Published:** 2026-03-12

**Authors:** Wolfram A. Bosbach, Marie S. Heide, Nasir Gözlügöl, Dana Fatemeh, Foroud Aghapour Zangeneh, David Ventura, Philipp Schindler, Wolfgang Roll, Franziska Strunz, Federico Caobelli, Kuangyu Shi, Ali Afshar-Oromieh, Axel Rominger, Robert Seifert

**Affiliations:** 1 Department of Nuclear Medicine, Inselspital, Bern University Hospital, University of Bern, Bern, Switzerland; 2 Department of Nuclear Medicine, University Hospital of Münster, Münster, Germany; 3 Department of Radiology, University Hospital of Münster, Münster, Germany

**Keywords:** AI in clinical practice, automation, LLM, medical technology, melanoma FDG PET/CT

## Abstract

**Purpose:**

Oncological patients regularly undergo PET/CT re-staging, which requires a report that outlines their current disease status and highlights relevant changes compared to the previous PET/CT. Large language models (LLMs) may be helpful with documentation in the future. This study is a pilot on LLM performance, focusing on test–retest stability and reproducibility.

**Methods:**

Three textbook melanoma follow-up cases of increasing complexity (involving one to eight organs) were selected. From standardized text-only prompts (no imaging data), follow-up reports were written by GPT-4o, Claude Sonnet 4 (each producing three independent revisions), and three nuclear medicine residents. This yielded nine reports per case (27 in total). Six blinded nuclear medicine experts (three internal, three external) performed test–retest evaluations of report quality and authorship identification.

**Results:**

The cosine similarity analysis revealed high intra-case coherence (mean: 0.599–0.727) regardless of authorship. The external human readers consistently rated reports higher than the internal human readers. The LLM-generated reports received comparable or superior ratings to human reports, with Claude achieving the highest external reader scores (mean 0.926, standard deviation 0.263, on a 0–1 scale). Human performance declined with case complexity, while Claude, in particular, improved. The external readers significantly preferred the LLM impressions (Fisher’s exact test, *p* = 0.005). Neither the human nor LLM readers reliably identified authorship (balanced accuracy 0.343–0.500).

**Conclusion:**

In this pilot, blinded expert evaluation demonstrated that current LLMs can generate reports for melanoma [^18^F]fluorodeoxyglucose PET/CT of comparable quality to human-authored reports from text prompts in this study. High test–retest stability was obtained. Larger future studies will be required to confirm these findings.

## Introduction

While demand for medical imaging, particularly in oncology, is expected to rise substantially, advances in artificial intelligence (AI) and large language models (LLMs) offer promising solutions to improve productivity and efficiency (1, 2). LLMs are especially promising because they can support clinical staff in documentation and administration. This is important, as personnel cost accounts for more than 55% of hospital expenditures in the United States, for example (3). A substantial portion of staff time is currently dedicated to administrative tasks (4), an area where LLMs can provide meaningful support and streamline workflows (5, 6). In what form and to what extent LLMs will contribute to medical processes remains to be seen (7, 8).

Currently, the documentation required by nuclear medicine specialists for positron emission tomography/computed tomography (PET/CT) demands substantial staff resources. Human doctors view the acquired image data and manually convert the contained information into text data, i.e., the PET/CT report, which is then sent to the referring physician. For this task, they often rely on only basic technological aids such as speech recognition software. Under the condition that they function adequately, LLMs can potentially be well-suited to assist in this process by generating PET/CT report texts, thereby reducing the workload of clinical staff. The optimal format for future PET/CT reports is subject to research itself, as evolving technologies and clinical needs continue to shape reporting standards (9, 10). A recent pilot study demonstrated that integrating retrieval-augmented LLMs with extensive PET/CT imaging report databases can significantly enhance clinicians’ ability to reference similar cases and generate more accurate differential diagnoses in nuclear medicine reporting (11). Domain-adapted LLMs have demonstrated substantial improvements in classifying PET/CT lymphoma reports, achieving up to 77.4% accuracy in predicting five-point Deauville scores (12), surpassing vision-only models and matching multimodal systems (13). Fine-tuned LLMs such as PEGASUS (14) have demonstrated the ability to generate personalized and clinically acceptable impressions for whole-body PET reports, achieving an 89% clinical acceptability rate and utility scores comparable to those of human physicians (15). A recurring research topic is the ability of LLMs to pass (Board) examinations. Due to the high inter-rater comparability when answering closed questions, these studies allow an insight into the evolution of LLMs over the last couple of years, which have seen steady system improvements (16, 17). Adequate review of LLM-generated texts remains a key aspect in LLM assessment, requiring expert readers—human or LLM-based—and appropriate quantitative evaluation methods (18). Thus, the above-mentioned research follows the idea of the first AI thinkers (19) of automating activities through the use of computers.

[^18^F]Fluorodeoxyglucose ([^18^F]FDG) PET/CT offers substantial clinical benefits for patients with melanoma and holds strong promise for future advancements in oncologic care (20, 21). Patients often undergo re-staging to assess their response to therapy or detect relapse early, which results in the need to write PET reports for follow-up examinations, especially relevant in patients with melanoma. This task is generally twofold: first, the changes in PET/CT findings between the examinations need to be objectified by measurement or description by a nuclear medicine physician. Second, the findings need to be integrated into a structured report that compares the findings with the previous results and is understandable by the referring physician.

**Figure 1:**
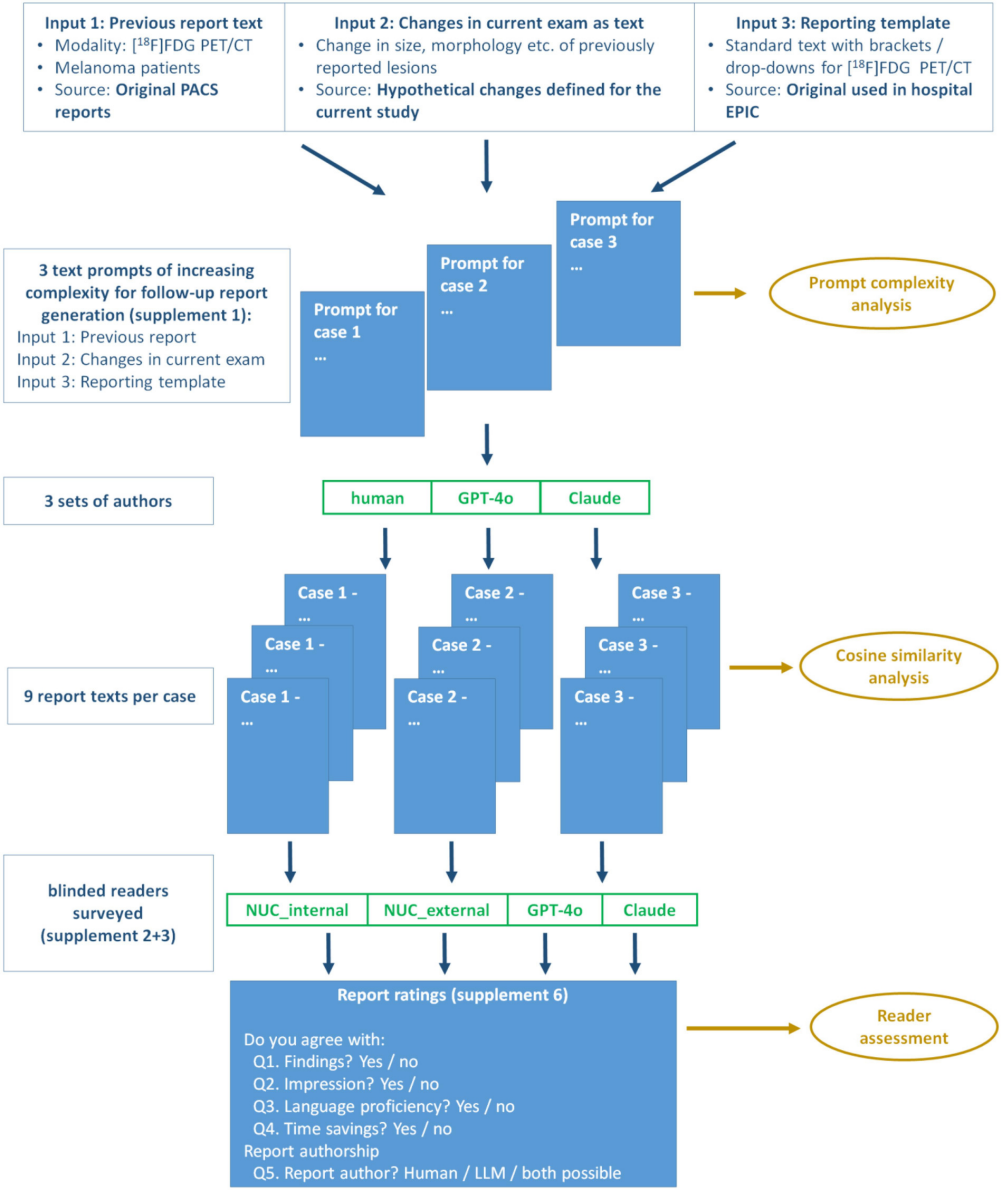
Project steps for report generation as a follow-up examination to a previous melanoma [^18^F]FDG PET/CT.

In this study, we aim to make a contribution to our rapidly evolving field by testing the ability of two currently available LLMs to write adequate [^18^F]FDG PET/CT reports for patients with melanoma from text-based prompts. Specifically, we investigate model stability and test–retest reproducibility, with nine revisions of text generation for each of three textbook melanoma cases. Participating authors (human authors and LLMs) received defined text-only prompts containing the findings of a melanoma [^18^F]FDG PET/CT examination (Figure 1). To increase the level of required expertise, these findings had to be interpreted as a follow-up in conjunction with a previously provided [^18^F]FDG PET/CT examination. The assessment by blinded readers follows a Python methodology previously used for data extraction from imaging cases (22).

## Methods and materials

Figure 1 shows the project steps defined for the current study. Three genuine cases for the [^18^F]FDG melanoma studies were selected from the picture archiving and communication system (PACS). Based on those, follow-up report texts were generated by human authors and LLMs.

### PACS input data and prompt definition

Three genuine melanoma [^18^F]FDG reports were selected from the PACS as typical representations of this tumor entity. Imaging was performed on a Biograph Vision Quadra PET/CT (Siemens Healthineers, Knoxville, TN, USA). The texts served as the previous examination. After anonymization (removal of the patient’s name and changing of dates), one prompt per case was created.

The prompts contained the following:

1. the general report template for [^18^F]FDG PET/CT,
2. the anonymized report text of the previous examination, and
3. hypothetical changes to the previous examination.

The hypothetical changes were defined by the study authors. Case selection and prompt definition aimed to provide the study with three typical cases of melanoma PET/CT of increasing complexity. To achieve increasing complexity, the number of affected organs increased from 1 (case 1) to 8 (case 3) and the word count of the defined prompts increased from 415 (case 1) to 826 (case 3, Table 1). This study relied exclusively on report text. No image data were included.

**Table 1:** Complexity of prompts for report generation and cosine similarity between generated report texts (Figure 2).

Case	Prompt		Cosine similarity between generated reports					
	Organs involved	Word count	Mean	Median	Min	Max	95% CI	SD
1	1	415	0.688	0.704	0.486	0.861	0.535–0.814	0.088
2	5	686	0.599	0.579	0.443	0.811	0.464–0.790	0.092
3	8	826	0.727	0.748	0.526	0.940	0.530–0.926	0.100
Inter-case	—	—	0.331	0.328	0.262	0.422	0.274–0.402	0.033

### [^18^F]FDG PET/CT report text generation

The three prompts for report generation for cases 1–3 were given to two current open-access LLMs. The first LLM was ChatGPT (model GPT-4o) (23) and the second LLM was Claude Sonnet 4 (24). Both LLMs were run for three revisions on each of the three text generation prompts to compensate for statistical variations in the LLM answering patterns. In addition, three final-year nuclear medicine residents were given the prompts for report generation for cases 1–3. The three residents completed the same tasks for text generation. Their texts serve as a reference group in the following analysis. In total, 27 texts were generated.

To prevent bias in the blinded review, one distinct formatting feature was removed. The “•” bullet symbol originally used by Claude was replaced by “-” prior to evaluation to avoid identification. An English translation of a sample text from Claude is provided in Table 2. Supplementary material S1 contains the prompt for report text generation.

**Table 2:** Example of a report text generated in German and translated into English for case 3, written by Claude and redacted for anonymization.

English translation: case 3, author = Claude, iteration 3, redacted version for anonymization
Clinical Information and Question: Metastatic malignant melanoma [XXX]. Staging PET-CT dated XXX Device: Siemens Biograph Vision Quadra Technique: Whole-body PET including low-dose CT, XXX minutes after administration via an intravenous access in the right hand Blood glucose: XXX mmol/L Premedication: none Radiotracer: XXX MBq [^18^F]FDG Findings: PET-CT from XXX available for comparison. Head/Neck: Normal appearance of the imaged neurocranium with physiological tracer uptake. No evidence of suspicious FDG-avid cervical lymph nodes. Thorax: Newly appearing, extensive, exophytic, highly FDG-avid soft tissue thickening anterolateral left, up to [XXX] wide (SUVmax [XXX]), confluent with previous lesions on the left lateral thoracic wall. Newly appearing, FDG-intense lymphadenopathy axillary left (3 lymph nodes, up to [XXX], SUVmax [XXX]) as well as along the left internal mammary artery (2 nodes up to [XXX]). No suspicious mediastinal or hilar lymph nodes. Increasingly extensive, metabolically active consolidations in both lungs, right-predominant peribronchial in the upper and lower lobes, and newly in the left upper lobe (SUVmax [XXX]). Imaging consistent with immune-related pneumonitis […]. Progression of atypical FDG uptake in the right atrium extending to the interventricular septum, SUVmax currently [XXX] (previously [XXX]). No pleural or pericardial effusion.[XXX] Abdomen: Significant metabolic and morphologic progression with now multiple, partially confluent FDG-avid liver lesions (SUVmax up to [XXX]), newly also in the caudate lobe and segment IVb. Several lesions with central photopenia, consistent with necrotic areas. Increase in size and FDG activity of both adrenal metastases (right [XXX]; left [XXX]; previously [XXX]). No evidence of bleeding or capsule rupture. Physiological tracer excretion via both kidneys. No free air or fluid. Skeleton/Extremities: Widespread involvement with now markedly more sclerotic lesions, especially in the sacrum and C1 vertebra. SUVmax in bone lesions tends to be decreasing (e.g., [XXX] vs. previous study [XXX]), consistent with partial metabolic remission. Status post dorsal spondylodesis of the thoracolumbar junction with cement augmentation. Status post left hip prosthesis. No new fractures or fracture-prone areas. Newly appearing, FDG-avid nodular lesion intramuscular in the left gluteus medius (SUVmax [XXX]). Impression: – Significant tumor progression of metastatic malignant melanoma with pronounced hepatic, adrenal, and cutaneous/subcutaneous metastases, as well as newly appearing axillary and paramammary lymphadenopathy left – Partial metabolic remission of bone metastases despite morphologically increasing sclerosis – Suspicion of cardiac metastasis vs. immune-mediated myocarditis in the context of progressive FDG uptake in the right atrium extending to the interventricular septum – Immune-related pneumonitis cannot be clearly distinguished from pulmonary tumor infiltration – further follow-up and interdisciplinary evaluation recommended

### Reader assessment

The generated 27 report texts for the three defined cases were assessed by blinded humans and by blinded LLM readers. Three internal nuclear medicine experts (NUC_internal) and three external nuclear medicine experts (NUC_external) read and graded the 27 report texts. For this purpose, the texts were provided in an online survey for the human readers (https://www.limesurvey.org). The 27 texts were shown in three blocks of 9, case by case. Within each block of nine, the order of the texts was randomized per reader. The readers were all board-certified nuclear medicine specialists/radiologists with a mean work experience of 10.5 ± 4.9 years [mean ± standard deviation (SD)].

In addition, GPT-4o (23) and Claude (24) were given the texts. Three revisions were obtained due to the already mentioned statistical variations of LLMs. The corresponding prompt for using the LLMs as readers is given in Supplementary material S2.

All readers, human and LLM, had to answer the following five questions with defined answering options for each of the 27 texts:

Do you agree with

*Q1.* Findings? Yes/no
*Q2.* Impression? Yes/no
*Q3.* Language proficiency? Yes/no
*Q4.* Time savings? Yes/no

Report authorship

*Q5.* Report author? Human/LLM/both possible

Questions 1–4 dealt with text quality. Question 5 asked the blinded reader about the text's authorship. Questions 1–4 were answered on a binary scale (positive/yes or negative/no). Although text quality can be assessed on a wider scale (25), larger scales often increase inter-reader variability, for example, due to differing interpretations of intermediate steps. The main interest in this study was whether a report could be sent to the referring physician or not. The readers were therefore asked to label each report as either suitable or unsuitable with respect to the quality requirements for a nuclear medicine report. This places the reader in the clinical workflow scenario where a senior doctor has to confirm a report text as final. Potential time savings were not quantified further; this study’s evaluation relied on positive/negative reader assessment for a hypothetical routine clinical application of the tested software. Question 4 relates to perceived rather than measured efficiency. Question 5 had a third option in case the reader was unable to define whether a text had been written by a human author or by an LLM. The obtained reader results are presented in Supplementary material S3.

The prompts for cases 1–3, the 27 generated report texts, and the reader survey were in German. All the internal and external human readers are native German speakers, board-certified in German, and practicing in their native language.

### Statistical analysis

**Figure 2:**
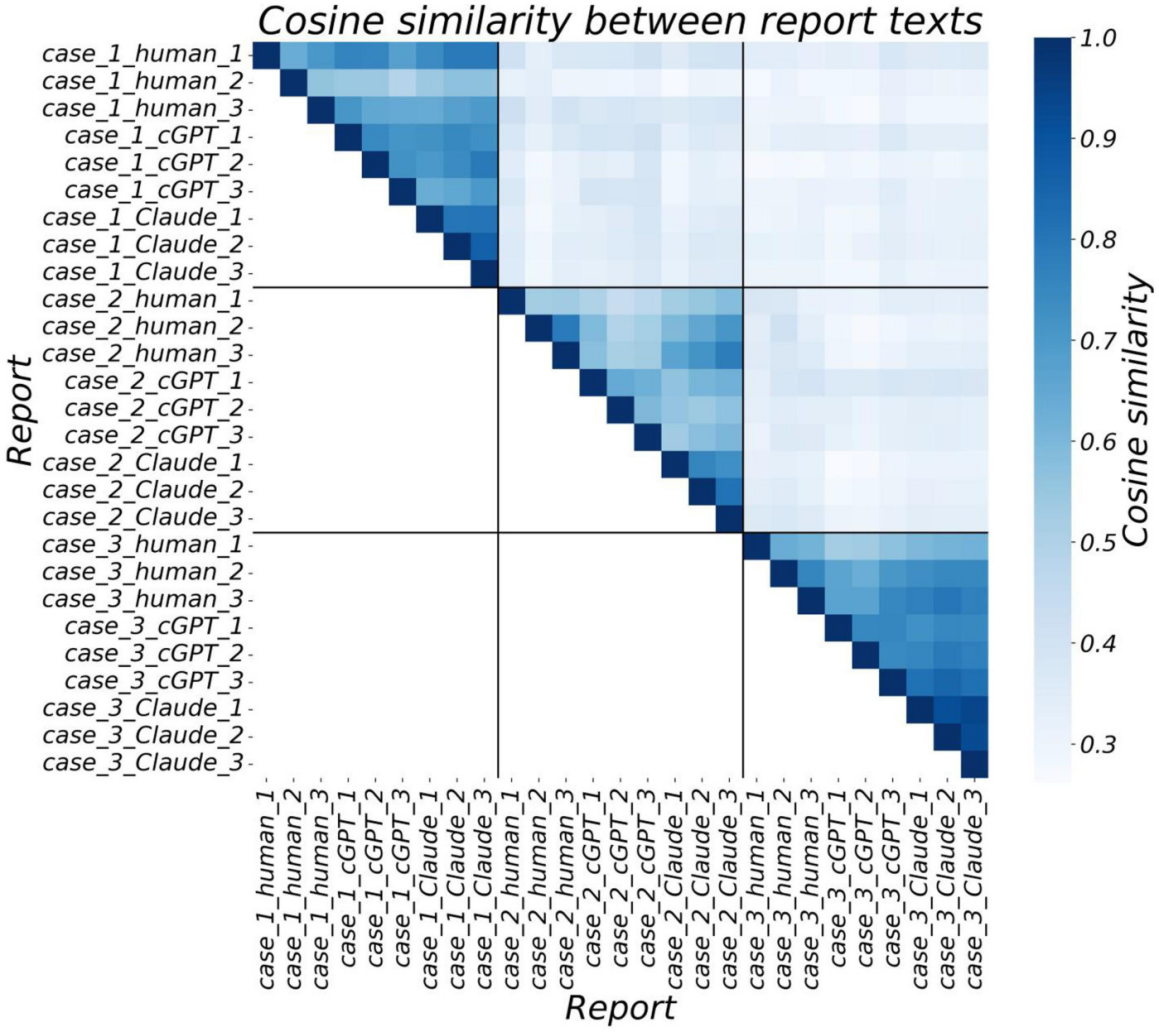
Cosine similarity on and above the main diagonal between the generated 27 report texts.

**Figure 3:**
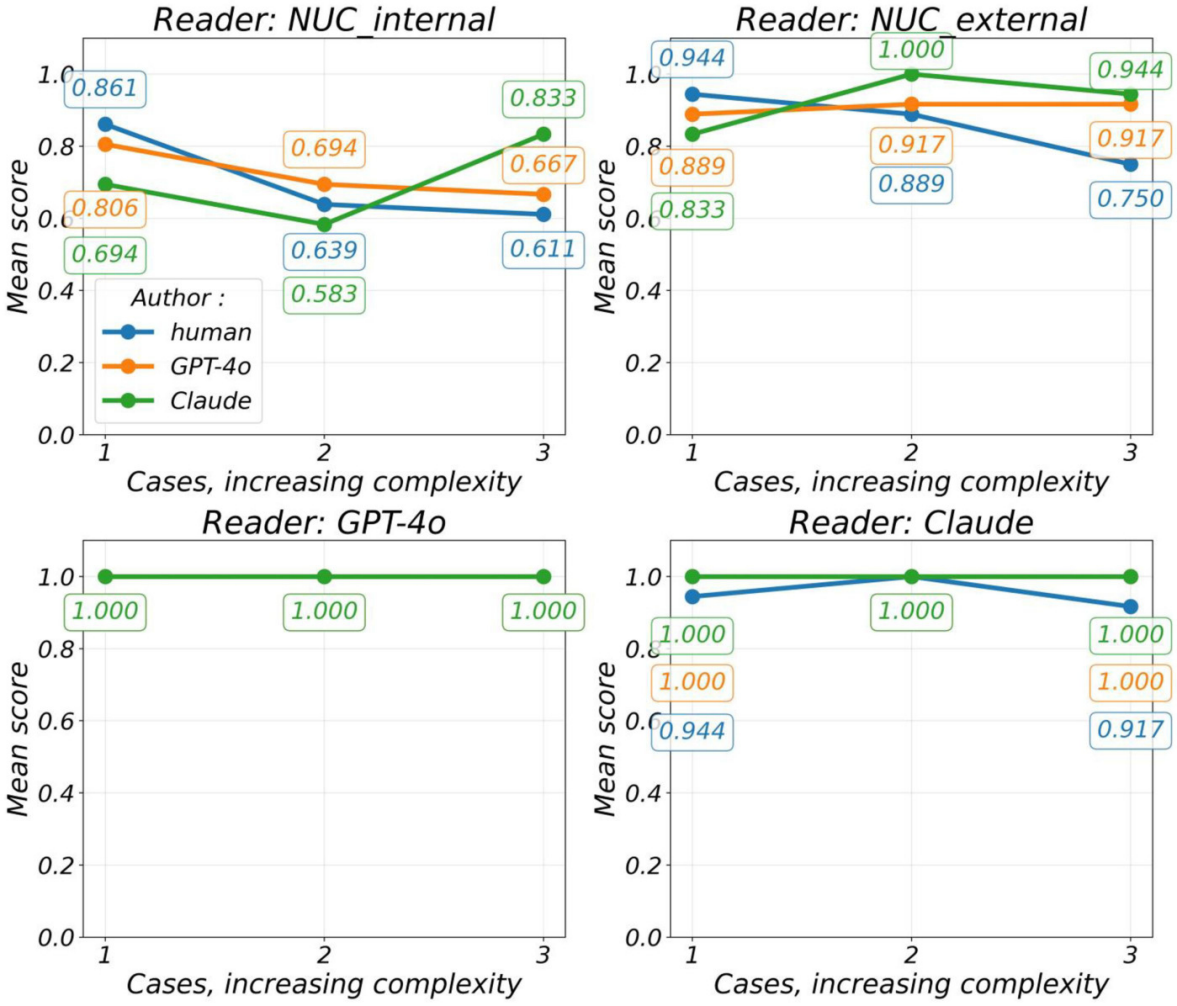
Mean score split by reader and by author, plotted over increasing case complexity (questions 1–4).

For the statistical analysis, a Python code was implemented and is provided with commented code lines as an open-access supplement (Supplementary material S4). Cosine similarity on the interval [−1,1] between the 27 generated reports texts was calculated after term frequency and inverse document frequency (Tfidf) vectorizing (26, 27) (Table 1 and Figure 2). For the quantified analysis of the reader results, the reader score was transformed into a numerical scale for questions 1–4 (yes = 1, no = 0, Table 3 and Figure 3). Details of the standard implementation of mean, median, 95% confidence interval (95% CI), SD, recall, etc., can be obtained from the Python code (Supplementary material S4). Cramer's V (28, 29) was implemented between text quality questions 1–4 and text complexity, and between text quality questions 1–4 and true author (Tables 3, 4). Cramer's V indicates for the interval (0–1) the degree by which two nominal variables are associated, independent of sample size: 0 = no association and 1 = perfect association. The interpretation of Cramer's V follows the framework offered by Cohen (29) for effect size on categorical data: small: >0.10, medium: >0.30, large: >0.50. This study interprets this further, with very small <0.1 and none if 0.

**Table 3:** Text quality assessment by readers (questions 1–4) and the influence of case complexity (Figure 3).

Text quality					
True author	Mean ± SD	NUC_internal	NUC_external	GPT-4o	Claude
Human	Q1: findings	0.667 ± 0.480	0.852 ± 0.362	1.000 ± 0.000	1.000 ± 0.000
GPT-4o		0.741 ± 0.447	0.852 ± 0.362	1.000 ± 0.000	1.000 ± 0.000
Claude		0.741 ± 0.447	0.889 ± 0.320	1.000 ± 0.000	1.000 ± 0.000
Human	Q2: impression	0.815 ± 0.396	0.704 ± 0.465	1.000 ± 0.000	1.000 ± 0.000
GPT-4o		0.926 ± 0.267	0.963 ± 0.192	1.000 ± 0.000	1.000 ± 0.000
Claude		0.778 ± 0.424	0.926 ± 0.267	1.000 ± 0.000	1.000 ± 0.000
Human	Q3: language	0.778 ± 0.424	1.000 ± 0.000	1.000 ± 0.000	0.815 ± 0.396
GPT-4o		0.593 ± 0.501	0.963 ± 0.192	1.000 ± 0.000	1.000 ± 0.000
Claude		0.741 ± 0.447	0.926 ± 0.267	1.000 ± 0.000	1.000 ± 0.000
Human	Q4: time saving	0.556 ± 0.506	0.889 ± 0.320	1.000 ± 0.000	1.000 ± 0.000
GPT-4o		0.630 ± 0.492	0.852 ± 0.362	1.000 ± 0.000	1.000 ± 0.000
Claude		0.556 ± 0.506	0.963 ± 0.192	1.000 ± 0.000	1.000 ± 0.000
Human	Combined Q1–4	0.704 ± 0.459	0.861 ± 0.347	1.000 ± 0.000	0.954 ± 0.211
GPT-4o		0.722 ± 0.450	0.907 ± 0.291	1.000 ± 0.000	1.000 ± 0.000
Claude		0.704 ± 0.459	0.926 ± 0.263	1.000 ± 0.000	1.000 ± 0.000
Cramer's V: text quality to case complexity					
True author = human	Q1: findings	0.509: large	0.390: medium	0.000: none	0.000: none
	Q2: impression	0.357: medium	0.303: medium	0.000: none	0.000: none
	Q3: language	0.218: small	0.000: none	0.000: none	0.357: medium
	Q4: time saving	0.000: none	0.289: small	0.000: none	0.000: none
	Combined Q1–4	0.245: small	0.236: small	0.000: none	0.165: small
Cramer's V: text quality to case complexity					
True author = LLM	Q1: findings	0.431: medium	0.156: small	0.000: none	0.000: none
	Q2: impression	0.195: small	0.343: medium	0.000: none	0.000: none
	Q3: language	0.167: small	0.343: medium	0.000: none	0.000: none
	Q4: time saving	0.141: small	0.090: very small	0.000: none	0.000: none
	Combined Q1–4	0.116: small	0.148: small	0.000: none	0.000: none

**Table 4:** Identification of the true author (question 5, Figure 4) and the influence of true author on reader assessment with χ^2^/FET significance testing after FDR correction.

True author					
Question 5	Performance	NUC_internal	NUC_external	GPT-4o	Claude
	Recall human	0.407	0.370	0.000	0.000
	Recall LLM	0.278	0.370	1.000	0.833
	Balanced accuracy	0.343	0.370	0.500	0.417
	Uncertainty rate	0.469	0.284	0.000	0.173
Cramer's V: text quality to true author					
	Q1: findings	0.077: very small	0.025: very small	0.000: none	0.000: none
	Q2: impression	0.048: very small	0.331: medium	0.000: none	0.000: none
	Q3: language	0.115: small	0.139: small	0.000: none	0.363: medium
	Q4: time saving	0.035: very small	0.029: very small	0.000: none	0.000: none
	combined Q1–4	0.010: very small	0.087: very small	0.000: none	0.177: small
*P*-value of χ^2^ test of independence [FET if min(cells) <5], data split by true author					
	Q1: findings	0.663: χ^2^	1.000: FET	1.000: FET	1.000: FET
	Q2: impression	0.915: χ^2^	0.005: FET^a^	1.000: FET	1.000: FET
	Q3: language	0.439: χ^2^	0.547: FET	1.000: FET	0.003: FET^a^
	Q4: time saving	0.937: χ^2^	1.000: FET	1.000: FET	1.000: FET

a Significance after FDR correction marked.

The chi-squared (χ^2^) test of independence (30) between the reader results obtained for quality questions 1–4, split by true author (human or LLM), was used (Table 4). Fisher’s exact test (FET) (31) was applied if one cell of the contingency matrix had a value <5. The Benjamini–Hochberg procedure for controlling the false discovery rate (FDR) was applied to correct raw *p*-values, i.e., counterbalancing inflation of Type I error due to multiple hypothesis testing (32).

Inter-reader reliability (IRR) was calculated using the irrCAC package (33). Gwet and Fleiss’ kappa were extracted together with their corresponding *p*-value (Table 5). Their interpretation regarding the LLM responses is discussed below. The IRR *p*-value in the irrCAC implementation tests whether the null hypothesis (H0), i.e., there is no agreement between readers beyond what is expected by chance, can be rejected. Gwet is especially useful for imbalanced data (34). Gwet and Fleiss take both values in the interval [−1,1]. The following framework for the interpretation of numerical IRR has been offered by Landis and Koch (35): <0: poor, >0: slight, >0.2: fair, >0.4: moderate, >0.6: substantial, >0.8: almost perfect, and : perfect.

**Table 5:** IRR by Gwet and Fleiss’ kappa.

IRR	Variable	NUC_internal	NUC_external	GPT-4o	Claude
Gwet	Coefficient	0.400	0.670	1.000	0.961
	Interpretation	Fair	Substantial	Perfect	Almost perfect
	*p*-value	0.000	0.000	0.000	0.000
Fleiss’ kappa	Coefficient	0.165	0.371	1.000	0.901
	Interpretation	Slight	Fair	Perfect	Almost perfect
	*p*-value	0.000	0.000	0.000	0.000

## Results

### Cosine similarity

The cosine similarity between the 27 generated report texts reached the global maximum (perfect 1 = absolute possible maximum) on the main diagonal, comparing report texts to themselves (Figure 2). Above the main diagonal, three plateaus of triangular shape corresponding to cases 1–3 were obtained. The mean cosine similarity ± SD was between 0.599 ± 0.092 (case 1) and 0.727 ± 0.100 (case 3) for the intra-case comparisons (Table 1). The corresponding value dropped to 0.331 ± 0.033 for the inter-case comparisons. The range of cosine similarity from the intra-case calculation and the inter-case calculation did not overlap; the maximum inter-case value of 0.422 was less than the minimum intra-case value of 0.443.

### Report text quality by reader

The numerically transformed reader assessments (Table 3) from NUC_external were consistently equal to or higher than those from NUC_internal across all questions and authors. For the human-authored reports, the NUC_external scores ranged from a minimum of 0.704 ± 0.465 (Q2: impression) to a maximum of 1.000 ± 0.000 (Q3: language), while the NUC_internal scores ranged from a minimum of 0.556 ± 0.506 (Q4: time saving) to a maximum of 0.815 ± 0.396 (Q2: impression). For the reports authored by GPT-4o, the NUC_external scores ranged from a minimum of 0.852 ± 0.362 to a maximum of 0.963 ± 0.192, compared to a range of a minimum of 0.593 ± 0.501 to a maximum of 0.926 ± 0.267 for NUC_internal. Similarly, for the Claude-generated reports, the NUC_external scores ranged from 0.889 ± 0.320 to 0.963 ± 0.192, with the NUC_internal scores ranging from 0.556 ± 0.506 to 0.778 ± 0.424. The combined Q1–4 scores were also higher for NUC_external across all authors: human (0.861 ± 0.347 vs. 0.704 ± 0.459), GPT-4o (0.907 ± 0.291 vs. 0.722 ± 0.450), and Claude (0.926 ± 0.263 vs. 0.704 ± 0.459).

Across all questions and authors, GPT-4o assigned perfect scores (1.000 ± 0.000) in every case. This includes its evaluations of human-written reports and those generated by itself and Claude, with no variation across the different quality dimensions. In the answering data from GPT-4o, no discrimination between human- and AI-authored content was found.

Across the majority of the categories, Claude assigned perfect scores (1.000 ± 0.000) to all reports, indicating a generally high evaluation regardless of authorship. The only exception occurred in Q3 (language), where Claude rated human-authored reports slightly lower at 0.815 ± 0.396. Claude perceived minor limitations in human language use compared to AI-generated reports. The combined Q1–4 score for human-authored reports was 0.954 ± 0.211, slightly lower than the 1.000 ± 0.000 scores of both AI models.

### Influence of case complexity

The mean scores for text quality ratings split by readers and plotted over increasing case complexity are summarized in Figure 3. For the NUC_internal readers, human performance decreased with complexity (0.861–0.611), whereas Claude improved (0.694–0.833) and GPT-4o showed a slight decrease (0.806–0.667). Both NUC_internal and NUC_external rated case 1 as (minimum complexity) Human > GPT-4o > Claude, and case 3 (maximum complexity) as Claude > GPT-4o > human. Text authored by GPT-4o and Claude achieved perfect mean scores (1.000) across all cases when evaluated by GPT-4o and Claude as readers, indicating perfect agreement. In contrast, human-authored texts received partially lower scores from Claude, with mean scores of 0.944 and 0.917 for cases 1 and 3, respectively.

Cramer's V between text quality ratings and case complexity (cases 1–3) varied by question and true author (human vs. LLM, Table 3). For human-authored reports, the NUC_internal ratings showed the strongest associations, with effect sizes ranging from 0.000 (none) to 0.509 (large). The highest association was observed for Q1 (findings) (0.509, large), followed by Q2 (impression) (0.357, medium) and Q3 (time savings) (0.218, small). The NUC_external ratings also demonstrated medium-to-small associations, with Q1 (findings) (0.390, medium) and Q2 (impression) (0.303, medium) yielding the highest values. The combined Q1–4 scores showed small associations for both NUC_internal (0.245) and NUC_external (0.236). In contrast, for the LLM-authored reports, the effect sizes were consistently smaller. NUC_internal values ranged from 0.141 (small) to 0.431 (medium), with the highest association again observed for Q1 (findings) (0.431, medium). The NUC_external values were highest for Q2 and Q3 (0.343, medium), while all other associations remained small or very small. Ratings from GPT-4o and Claude showed no association (0.000) in the majority of cases, except for Claude's Q3 rating of human-authored reports (0.357, medium) and combined Q1–4 score (0.165, small).

### True author: identification by readers and significance testing

**Figure 4:**
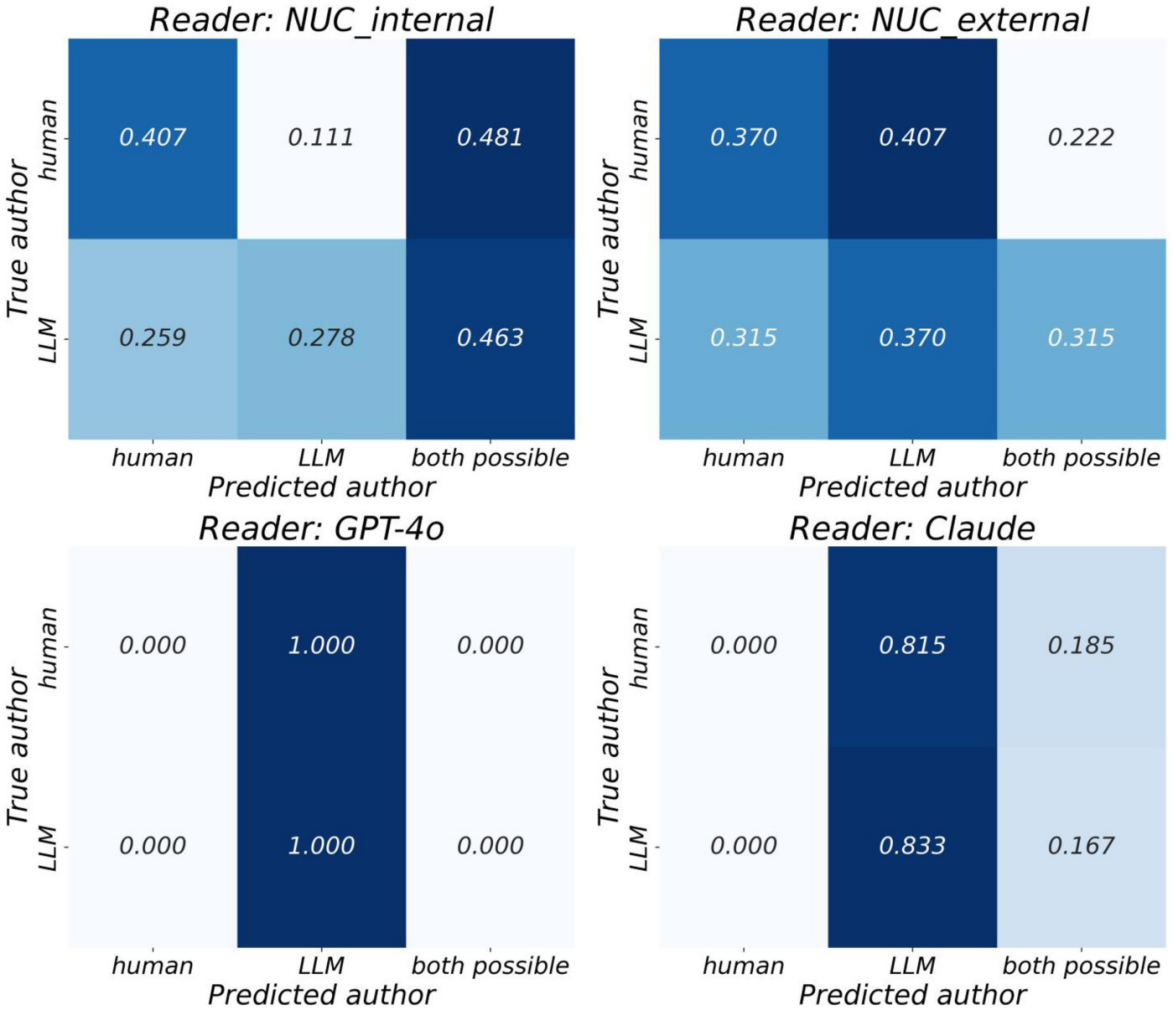
Confusion matrix by reader, true author over predicted author (question 5).

For Question 5, which asked readers to identify the true author, performance varied across reader types (Figure 4 and Table 4). The NUC_internal readers showed moderate recall for human-authored responses (0.407) but lower recall for LLM-authored responses (0.278), resulting in a balanced accuracy of 0.343. The uncertainty rate—cases labeled as “both possible”—was greater than for any other reader (0.469). The NUC_external readers demonstrated slightly more balanced performance, with equal recall for human and LLM texts (0.370 each), yielding a balanced accuracy of 0.370 and a lower uncertainty rate of 0.284. In contrast, GPT-4o, as a reader, exclusively predicted all reports as LLM-generated, resulting in a recall of 1.000 for LLM-authored texts but 0.000 for human-authored ones. This yielded a balanced accuracy of 0.500 and an uncertainty rate of 0.000. Claude, as a reader, similarly showed strong LLM identification (recall = 0.833) but failed to identify any human-authored texts (recall = 0.000), with a balanced accuracy of 0.417 and uncertainty rate of 0.173.

Cramer's V was used to assess the association between the text quality ratings (Questions 1–4) and the true author (human vs. LLM, Table 4). For the NUC_internal readers, the highest association between text quality and true author was observed in the time savings question (Q3) with a Cramer's V of 0.115 (small), while the lowest association was found for the combined Q1–4 score with 0.010 (very small). For the NUC_external readers, the highest association was noted for the impression question (Q2) with a Cramer's V of 0.331 (medium), and the lowest association was for findings (Q1) with 0.025 (very small). The other questions demonstrated small to very small effects. The Cramer’s V for the combined Q1–4 score was 0.087, classified as very small.

GPT-4o, as a reader, showed no association (Cramer's V = 0.000) across all the individual and combined questions. In contrast, Claude, as a reader, demonstrated a medium association for language proficiency (Q3), with a Cramer's V = 0.363, and a small overall association when combining all the questions (0.177), with no association for findings, impression, or time savings.

The χ^2^/FET test revealed no significance between text quality ratings and true author for the NUC_internal readers or GPT-4o (all *p* > 0.05, Table 4). The NUC_external readers showed a significant association for impression (Q2; FET *p* = 0.005, also significant after FDR correction). Claude, as a reader, showed significant associations for language (Q3; FET *p* = 0.003, also significant after FDR correction).

### Inter-rater reliability

Based on Gwet, agreement was fair for the NUC_internal readers (0.400), substantial for NUC_external (0.670), perfect for GPT-4o (1.000), and almost perfect for Claude (0.961), all with *p* < 0.05 (Table 5). Using Fleiss’ kappa, the NUC_internal readers showed slight agreement (0.165), NUC_external showed fair agreement (0.371), while GPT-4o (1.000) and Claude (0.901) demonstrated perfect and almost perfect agreement, respectively. All Fleiss’ kappa values were statistically significant (*p* < 0.05). Overall, IRR was highest for GPT-4o, followed closely by Claude, with both AI systems substantially more consistent than the human reader groups.

## Discussion

This study used LLMs to simulate the generation of PET/CT reports in melanoma follow-ups. Blinded reviewers (external clinicians, internal clinicians, and LLMs) evaluated reports from the perspective of a senior physician responsible for final sign-off, i.e., the approval step that releases the report to the referring physician.

### Clinical integration perspectives

Although this study did not evaluate clinical deployment directly, several practical pathways for integrating LLM-generated PET/CT follow-up reports into real-world workflows can be envisioned (19). A realistic early application would be a human–AI hybrid workflow, in which LLMs generate an initial structured draft that is reviewed, corrected, and signed off by a nuclear medicine physician (6, 36). Alternatively, LLMs may support structured reporting systems, helping to ensure linguistic consistency, completeness, and adherence to institutional templates. Another promising role may be quality-control support, where LLMs act as a secondary reader to highlight inconsistencies, missing key findings, or deviations from prior reports (22). Any clinical implementation will require clear governance, transparency of AI involvement, robust validation, and retained clinical responsibility with the human expert.

### Formal text similarity

It is established that text similarity metrics, such as cosine similarity, can reveal intra- and inter-model patterns in LLM outputs (37). In the present study, cosine similarity was highest for intra-case comparisons, regardless of a text's author. This suggests that both the evaluated LLMs, GPT-4o (23) and Claude Sonnet 4 (24), can successfully generate reports that exhibit a distinct level of semantic and structural similarity comparable to that of reports authored by nuclear medicine physicians. However, high cosine similarity should not be interpreted as indicating clinically identical meaning, which is why human internal and external readers are essential.

### Interpretation of IRR/LLM stability

Despite being blinded to authorship, both GPT-4o and Claude consistently rated all reports—whether human- or LLM-authored—with uniformly high scores. This lack of discrimination suggests a possible intrinsic bias in LLM evaluators toward stylistic or structural features typical of AI-generated text. Recent work has shown that LLMs often align more closely with the linguistic patterns of their own outputs, even when assessing content produced by other sources, raising concerns about self-reinforcing evaluation loops in LLM-based systems (38). Due to the limited insights into LLM decision-making processes, there is debate whether Fleiss’ and Gwet’s coefficients (Table 5) represent true IRR measures in LLMs or rather provide an intra-rater test–retest assessment of model stability with possible memory components (39). Despite this uncertainty in interpretation, the high agreement coefficients demonstrate high model stability and absence of chaotic statistical variations, which is crucial for potential clinical applications.

In contrast, human readers displayed greater variability and differentiation (40), underscoring their continued value in nuanced quality assessments. Accordingly, the obtained IRR between blinded human readers varied from slight to fair for Fleiss’ kappa and fair to substantial for Gwet. This limited level of agreement between human readers has been found in other text quality studies (6, 25).

Whether the fluctuations in reader results, be it human or LLM readers, reflect genuine quality differentials or inter-rater noise is not answered in this study. Future study designs will benefit from the inclusion of an independent ground truth.

### Measurable effects on report quality from blinded report assessment

This study measured the performance of authors through quality assessment by blinded readers. Internal and external nuclear medicine specialists and the two LLMs graded the generated texts by answering closed (yes/no) questions on text quality. The survey asked the readers to take the role of a senior doctor who, in the clinical workflow, has to confirm a report text as final. In the assessments by the external and the internal human readers, the performance of human authors declined with increasing case complexity. In contrast, there was an observable trend that LLM texts were preferred at higher complexity levels, suggesting advantages in using AI in challenging cases. The limited sample size means that the observations should be seen as exploratory. If validated in the future, this would be consistent with evidence showing multimodal AI models can outperform human diagnoses in up to 85% of cases (41). Significant differences between human-authored texts and AI-authored texts were found by external human readers for Q2 (impression; *p* = 0.005, FET), and by Claude for Q3 (language; *p* = 0.003, FET, Table 4).

### True author indistinguishable to human and LLM readers

None of the readers, human or LLM, reliably identified the true author of the reports. Human readers showed low balanced accuracy and high uncertainty, while LLMs, despite being blinded, labeled none of the reports as human-generated. This highlights a broader challenge in authorship attribution for AI-generated clinical text and supports previous findings that LLMs lack reliable self-critique or author discrimination capabilities (38). However, the limited sample size means that the obtained results should be seen as exploratory.

### Importance of external evaluation

In our study, the NUC_external readers consistently assigned scores that were equal to or higher than those of the NUC_internal readers across all questions and author types. Similar patterns of performance consistency by external readers have been observed in radiology studies, where independent external readers offer consensus-based or majority interpretations that serve as surrogate standards. For instance, a multi-institutional investigation of body CT interpretation involved 31 external radiologists across 22 centers; these external assessments helped define a surrogate “reference standard,” against which primary internal readings were compared, to evaluate interpretation variability and generalizability (42). This underscores the potential value of external evaluations in obtaining robust, calibrated assessments of report quality, particularly in contexts like ours where internal and external reader scores diverge.

### Study limitations

The internal validity of this study is constrained by the small number of original cases (*n* = 3). While each case was deliberately designed as a textbook melanoma follow-up scenario and generated 9 independent report versions (yielding 27 texts), the limited number of base cases restricts the precision with which effect sizes could be estimated. The observed differences between the authors and readers, therefore, need to be interpreted as exploratory rather than definitive. Nevertheless, the consistent directionality of several findings, such as IRR levels (Table 5), suggests that the observed effects are not purely random, even if their magnitude cannot be robustly quantified.

External validity is limited by the deliberate case selection strategy. The three cases represent typical, well-defined melanoma follow-up scenarios of increasing complexity rather than a representative sample of the full clinical spectrum. Consequently, the results do not allow for direct generalization to all oncologic PET/CT follow-up examinations, rare edge cases, or institutions with substantially different reporting cultures. The intent of this study was not to establish population-level performance metrics or generalizable trends, but to evaluate model stability and reader perception under controlled and clinically realistic conditions. Future studies with larger and more heterogeneous case collections will be required to assess generalizability across diseases, institutions, and reporting styles.

Reproducibility is inherently limited by the nature of contemporary large language models. GPT-4o and Claude are systems that function as “black boxes” and may be modified, restricted, or withdrawn by their providers without notice. Indeed, the browser-based versions used in this study are no longer accessible in their original form. As a result, exact replication of the reported outputs is not guaranteed, independent of study design quality. This limitation applies not only to LLM-based experiments but increasingly to AI benchmarking in general. To mitigate this, all prompts, evaluation criteria, and analysis code are provided openly (Supplementary material S1–S4), allowing conceptual and methodological replication even if bit-wise reproducibility cannot be ensured. The observed trends should therefore be interpreted with caution, and future work will benefit from the inclusion of independent ground truth definitions and longitudinal benchmarking.

Our study did not assess actual clinical implementation. For the sake of complexity reduction, the current study's design included text-only processes (Figure 1). The reports were generated and evaluated in an experimental simulation, outside of clinical workflows. It remains unclear how LLM-generated reports would perform in real-world diagnostic or interdisciplinary settings, where reader context, time constraints, and responsibility for clinical decisions may affect perception and usability.

Our evaluation was limited to text-based prompts and report generation. No imaging data were used, and the LLMs did not interpret or “see” the PET/CT images. As such, this study does not assess whether LLMs can independently derive findings from medical images, but rather whether they can produce coherent and clinically acceptable follow-up reports based on structured textual input.

A further limitation is that all source data and report templates came from a single institution, reducing diversity in clinical styles and practices. All the evaluations were conducted in German, which may limit applicability to other languages. A sensitivity test related to increasing case complexity was performed (Figure 3); however, no sensitivity test was included that investigated the effect of minor input perturbations.

## Conclusion

The LLMs used in this study demonstrated high model stability. In the blinded reader assessment, they were marginally superior to human authors when tasked with [^18^F]FDG PET/CT melanoma follow-up report writing, with the external human readers preferring the LLM-generated reports in question 2. Claude preferred the language of LLM-generated reports (Question 3). Given the ongoing improvement of LLM performance and their computational speed, it appears possible that such systems will find their way into clinical applications in the future. The improvements that can be expected from the usage of AI systems are, however, not limitless; the reconstruction of magnetic resonance imaging (MRI) is one example (43, 44). It remains to be seen what the contribution of LLMs in the near future will be to the writing of nuclear medicine reports (5, 7). An increase in productivity would be in the best interest of patients, doctors, and hospital administrators alike (3), with the caveat that the quality of patient outcomes is maintained or even increased. Computers automatically writing complex PET/CT reports and interacting with human healthcare staff in hybrid processes would bring into reality the predictions of the first AI thinkers from the 1950s (19). Despite the promising results of this study, the deskilling of medical doctors due to computer processes appears not to be an immediate threat, at the moment at least (45).

## Data Availability

The original contributions presented in the study are included in the article/Supplementary Material, further inquiries can be directed to the corresponding authors.

## Supplementary material

The Supplementary Material for this article can be found online at: Zenodo, DOI: https://doi.org/10.5281/zenodo.18182814

Supplementary Material S1 Prompt for FDG PET/CT report generation, redacted for anonymization, German original/English translation (.txt).

Supplementary Material S2 LLM-rating prompt, German original/English translation (.txt).

Supplementary Material S3 Report ratings obtained in the blinded reader study (.xlsx).

Supplementary Material S4 Python code with comments for statistical analysis (.py).

## References

[1] Dell’Acqua F McFowland E III Mollick E Lifshitz-Assaf H Kellogg KC Rajendran S Navigating the Jagged Technological Frontier: Field Experimental Evidence of the Effects of AI on Knowledge Worker Productivity and Quality. Working Paper 24-013. (2023).

[2] Tang A Tam R Cadrin-Chênevert A Guest W Chong J Barfett J Canadian Association of Radiologists white paper on artificial intelligence in radiology. Can Assoc Radiol J. (2018) 69(2):120–35. 10.1016/j.carj.2018.02.002 29655580

[3] American Hospital Association. The Cost of Caring: Challenges Facing America’s Hospitals in 2025 (2025). p. 1–6. Available online at: https://www.aha.org/system/files/media/file/2025/04/The-Cost-of-Caring-April-2025.pdf (Accessed February 19, 2026).

[4] Woolhandler S Himmelstein DU. Administrative work consumes one-sixth of U.S. physicians’ working hours and lowers their career satisfaction. Int J Health Serv. (2014) 44(4):635–42. 10.2190/HS.44.4.a 25626223

[5] Guo L Tahir AM Zhang D Wang ZJ Ward RK. Automatic medical report generation: methods and applications. *arXiv* [Preprint]. *arXiv:2408.13988 [cs.CV]* (2024).

[6] Bosbach WA Clement C Strunz F Aghapour Zangeneh F Gözlügöl N Bregenzer CM Automation of 99mTc mercaptoacetyltriglycine (MAG3) report writing using a vision language model. EJNMMI Res. (2025) 15(142):1–10. 10.1186/s13550-025-01323-6 41324797 PMC12686326

[7] Alberts IL Mercolli L Pyka T Prenosil G Shi K Rominger A Large language models (LLM) and ChatGPT: what will the impact on nuclear medicine be? Eur J Nucl Med Mol Imaging. (2023) 50(6):1549–52. 10.1007/s00259-023-06172-w 36892666 PMC9995718

[8] Hirata K Matsui Y Yamada A Fujioka T Yanagawa M Nakaura T Generative AI and large language models in nuclear medicine: current status and future prospects. Ann Nucl Med. (2024) 38(11):853–64. 10.1007/s12149-024-01981-x 39320419 PMC11813999

[9] Naik SS Hanbidge A Wilson SR. Radiology reports: examining radiologist and clinician preferences regarding style and content. Am J Roentgenol. (2001) 176(3):591–8. 10.2214/ajr.176.3.1760591 11222186

[10] Niederkohr RD Greenspan BS Prior JO Schod¨er H Seltzer MA Zukotynski KA Reporting guidance for oncologic 18F-FDG PET/CT imaging. J Nucl Med. (2013) 54(5):756–61. 10.2967/jnumed.112.112177 23575994

[11] Choi H Lee D Kang YK Suh M. Empowering PET imaging reporting with retrieval-augmented large language models and reading reports database: a pilot single center study. Eur J Nucl Med Mol Imaging. (2025) 52(7):2452–62. 10.1007/s00259-025-07101-9 39843863 PMC12119711

[12] Barrington SF Kluge R. FDG PET for therapy monitoring in Hodgkin and non-Hodgkin lymphomas. Eur J Nucl Med Mol Imaging. (2017) 44(Suppl 1):S97–110. 10.1007/s00259-017-3690-8 PMC5541086 28411336

[13] Huemann Z Lee C Hu J Cho SY Bradshaw TJ. Domain-adapted large language models for classifying nuclear medicine reports. Radiol Artif Intell. (2023) 5(6):e220281. 10.1148/ryai.220281 38074793 PMC10698610

[14] Zhang J Zhao Y Saleh M Liu PJ. PEGASUS: pre-training with extracted gap-sentences for abstractive summarization. arXiv [Preprint]. abs/1912.08777 (2020). 10.48550/arXiv.1912.08777

[15] Tie X Shin M Pirasteh A Ibrahim N Huemann Z Castellino SM. Personalized impression generation for PET reports using large language models. J Imaging Informatics Med. (2024) 37(2):471–88. 10.1007/s10278-024-00985-3 PMC11031527 38308070

[16] Ting YT Hsieh TC Wang YF Kuo YC Chen YJ Chan PK Performance of ChatGPT incorporated chain-of-thought method in bilingual nuclear medicine physician board examinations. Digit Health. (2024) 10(2):1–10. 10.1177/20552076231224074 PMC10771043 38188855

[17] Oumano MA Pickett SM. Comparison of large language models’ performance on 600 nuclear medicine technology board examination–style questions. J Nucl Med Technol. (2025) 53(3):262–7. 10.2967/jnmt.124.269335 40345820

[18] Ohde JW Rost LM Overgaard JD. The burden of reviewing LLM-generated content. NEJM AI. (2025) 2(2):8–11. 10.1056/aip2400979

[19] McCarthy J Minsky ML Rochester N Shannon CE. A Proposal for the Dartmouth Summer Research Project on Artificial Intelligence [Internet] (1955). p. 1–13. Available online at: http://jmc.stanford.edu/articles/dartmouth/dartmouth.pdf (Accessed February 19, 2026).

[20] Alberts I Hünermund JN Prenosil G Mingels C Bohn KP Viscione M Clinical performance of long axial field of view PET/CT: a head-to-head intra-individual comparison of the Biograph Vision Quadra with the Biograph Vision PET/CT. Eur J Nucl Med Mol Imaging. (2021) 48:2395–404. 10.1007/s00259-021-05282-7 33797596 PMC8241747

[21] Narendra R Warissara J Bosbach WA Chen Y Penner JL Sari H Total body PET/CT: clinical value and future aspects of quantification in static and dynamic imaging. Semin Nucl Med. (2024) 55(1):98–106. 10.1053/j.semnuclmed.2024.11.004 39616013

[22] Bosbach WA Schoeni L Beisbart C Senge JF Mitrakovic M Anderson SE Evaluating the diagnostic accuracy of ChatGPT-4.0 in classifying multimodal musculoskeletal masses: a comparative study with human raters. RöFo. (2025). 10.1055/a-2594-7085 40461006

[23] OpenAI Inc. ChatGPT (4o) [Large language model] [Internet]. (2025). Available online at: https://chat.openai.com (accessed August 5, 2025).

[24] Anthropic PBC. Claude Sonnet 4 [Large language model] [Internet]. (2025). Available online at: https://www.anthropic.com (accessed August 5, 2025).

[25] Bosbach WA Senge JF Nemeth B Omar SH Mitrakovic M Beisbart C Ability of ChatGPT to generate competent radiology reports for distal radius fracture by use of RSNA template items and integrated AO classifier. Curr Probl Diagn Radiol. (2023) 53(1):102–10. 10.1067/j.cpradiol.2023.04.001 37263804

[26] cosine_similarity [Internet]. scikit-learn 1.6.1 documentation (2025). Available online at: https://scikit-learn.org/stable/modules/generated/sklearn.metrics.pairwise.cosine_similarity.html (accessed March 16, 2025).

[27] TfidfVectorizer [Internet]. scikit-learn 1.6.1 documentation (2025). Available online at: https://scikit-learn.org/stable/modules/generated/sklearn.feature_extraction.text.TfidfVectorizer.html (accessed March 16, 2025).

[28] scipy.stats.contingency.association [Internet]. SciPy v1.16.0. (2025). Available online at: https://docs.scipy.org/doc/scipy/reference/generated/scipy.stats.contingency.association.html (accessed July 26, 2025).

[29] Cohen J. Statistical Power Analysis for the Behavioral Sciences. 2nd ed. New York, NY: Lawrence Erlbaum Associates (1988).

[30] scipy.stats.chi2_contingency [Internet]. SciPy v1.14.1 Manual. (2024). Available online at: https://docs.scipy.org/doc/scipy/reference/generated/scipy.stats.chi2_contingency.html (accessed September 1, 2024).

[31] scipy.stats.fisher_exact [Internet]. SciPy v1.16.1. (2025). Available online at: https://docs.scipy.org/doc/scipy/reference/generated/scipy.stats.fisher_exact.html (accessed August 9, 2025).

[32] statsmodels.stats.multitest.multipletests [Internet]. statsmodels 0.15.0 (+841. (2025). Available online at: https://www.statsmodels.org/dev/generated/statsmodels.stats.multitest.multipletests.html (accessed November 12, 2025).

[33] Gwet K Fergadis A. irrCAC—chance-corrected agreement coefficients [Internet]. (2023). Available online at: https://www.irrcac.readthedocs.io/en/latest/usage/usage_raw_data.html (accessed September 3, 2025).

[34] Wongpakaran N Wongpakaran T Wedding D Gwet KL. A comparison of Cohen’s kappa and Gwet’s AC1 when calculating inter-rater reliability coefficients: a study conducted with personality disorder samples. BMC Med Res Methodol. (2013) 13(1):1–7. 10.1186/1471-2288-13-61 23627889 PMC3643869

[35] Landis JR Koch GG. The measurement of observer agreement for categorical data. Biometrics. (1977) 33(1):159–74. 10.2307/2529310 843571

[36] Bosbach WA Schoeni L Senge JF Mitrakovic M Weber MA Dlotko P Novel artificial intelligence chest X-ray diagnostics: a quality assessment of their agreement with human doctors in clinical routine. RöFo. (2025) (in press). 10.1055/a-2772-7798 41558503

[37] Smith B Bouadjenek MR Kheya TA Dawson P Aryal S. A comprehensive analysis of large language model outputs: similarity, diversity, and bias. arXiv [Preprint]. arXiv:2505.09056 (2025):1–19. 10.48550/arXiv.2505.09056

[38] Panickssery A Bowman SR Feng S. LLM evaluators recognize and favor their own generations. *arXiv* [Preprint]. *arXiv:2404.13076 [cs.CL]* (2024).

[39] Klishevich E Denisov-Blanch Y Obstbaum S Ciobanu I Kosinski M. Measuring determinism in large language models for software code review. *arXiv* [Preprint]. *arXiv:2502.20747 [cs.SE]* (2025): 1–15.

[40] Panicek DM Hricak H. How sure are you, doctor? A standardized lexicon to describe the radiologists level of certainty. Am J Roentgenol. (2016) 207(1):2–3. 10.2214/AJR.15.15895 27065212

[41] Ruan C Huang C Yang Y. Comprehensive evaluation of multimodal AI models in medical imaging diagnosis: from data augmentation to preference-based comparison. *arXiv* [Preprint]. *arXiv:2412.05536 [eess.IV]* (2024).

[42] Ryan AF Semelka RC Molina PL Yonkers S Vaidean G. Evaluation of radiologist interpretive performance using blinded reads by multiple external readers. Invest Radiol. (2010) 45(4):211–6. 10.1097/RLI.0b013e3181d2ee97 20177390

[43] Bosbach WA Merdes KC Jung B Montazeri E Anderson SE Mitrakovic M Deep learning reconstruction of accelerated MRI: false positive cartilage delamination inserted in MRI arthrography under traction. Top Magn Reson Imaging. (2024) 33(1–3):e0313. 10.1097/RMR.0000000000000313 39016321

[44] Granstedt J Kc P Deshpande R Garcia V Badano A. Hallucinations in medical devices. *arXiv* [Preprint]. *arXiv:2508.14118 [eess.IV]* (2025). 10.48550/arXiv.2508.14118

[45] Duran LDD. Deskilling of medical professionals: an unintended consequence of AI implementation? G Filos. (2021) 2:1–13. 10.7413/1827-5834014

